# Numerical simulation of lead-free vacancy ordered Cs_2_PtI_6_ based perovskite solar cell using SCAPS-1D†

**DOI:** 10.1039/d3ra04176j

**Published:** 2023-08-01

**Authors:** Akfeen Amjad, Samina Qamar, Chengchen Zhao, Kalsoom Fatima, Muhammad Sultan, Zareen Akhter

**Affiliations:** a Department of Chemistry, Quaid-i-Azam University (QAU) Islamabad 45320 Pakistan zareenakhter@yahoo.com zareen_a@qau.edu.pk; b School of Chemistry, University of Glasgow Glasgow UK 2792248Z@student.gla.ac.uk; c Department of Physics, Kohsaar University Muree (KUM) Muree Pakistan mssatti79@yahoo.com (+92) 51-9269174

## Abstract

In recent years, vacancy-ordered halide double perovskites have emerged as promising non-toxic and stable alternatives for their lead-based counterparts in optoelectronic applications. In particular, vacancy ordered Cs_2_PtI_6_ has emerged as a star material because of its high absorption coefficient, band gap of 1.37 eV, and long minority carrier lifetime. Despite substantial experimental research on this new class of material, theoretical simulations of their device properties remain scarce. In this work, a novel n-i-p device architecture (FTO/SnO_2_/Cs_2_PtI_6_/MoO_3_/C) is theoretically investigated using a solar cell capacitance simulator (SCAPS-1D). Theoretical investigations are carried out in order to optimize the device performance structure by varying the perovskite and selective charge transport layer thickness, absorber and interface defect density, operating temperature, back contact, series and shunt resistance, respectively. The optimized device showed an impressive power conversion efficiency (PCE) of 23.52% at 300 K, which is higher than the previously reported values. Subsequent analysis of the device's spectral response indicated that it possessed 98.9% quantum efficiency (QE) and was visibly active. These findings will provide theoretical guidelines for enhancing the performance of Cs_2_PtI_6_-based photovoltaic solar cells (PSCs) and pave the way for the widespread implementation of environmentally benign and stable perovskites.

## Abstract 2

In recent years, vacancy-ordered halide double perovskites have emerged as promising non-toxic and stable alternatives for their lead-based counterparts in optoelectronic applications.

## Graphical abstract

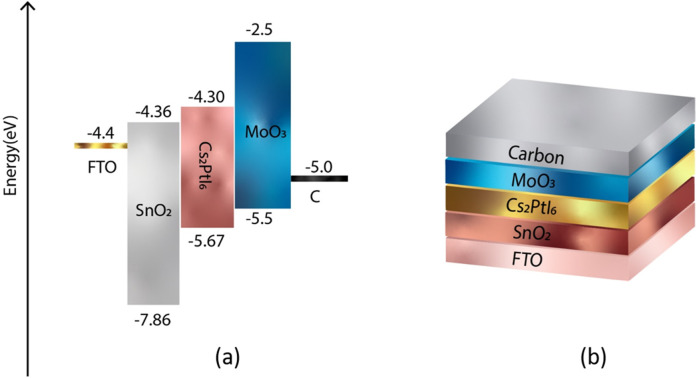

## 1 Introduction

In recent years, there has been an increasing interest in hybrid perovskite solar cells (HPSCs) due to their remarkable intrinsic properties, including lower production costs, higher carrier mobilities, tunable band gaps, long carrier diffusion lengths, and high absorption coefficient.^1–4^ In just over a decade, the PCE has dramatically increased from 3.3% to 25.7% due to the synergistic optimization of device interfaces. Full industrialization of HPSCs, in spite of having achieved a PCE of over 25% is trammeled by two main issues: Pb toxicity and instability. Organic ions methyl ammonium (MA^+^) and formamidinium (FA^+^) at the ‘A’ site of perovskite are unstable and degrade in atmospheric operational conditions.^5–10^ Consequently, it would be highly advantageous for device lifetimes and environmental and health considerations regarding production and reprocessing to seek out non-toxic and stable perovskites that sustain high efficiency. Materials such as (MA, Cs, Rb)_3_Sb_2_I_9_, Cs_2_PdBr_6_, Cs_2_AgBiBr_6_, Cs_2_AgBiI_6_, Cs_2_TiBr_6_, AgBiI_4_, CsGe_0.5_Sn_0.5_I_3_, and Cs_2_Au_2_I_6_ have been explored as lead-free alternatives, but their performance for photovoltaic applications has been disappointing.^11–21^

Finding stable, non-toxic, and highly efficient perovskites has proven to be an enormous problem to date. In recent years, A_2_BX_6_ (A=Cs; B=Te, Sn, Pt, Ti; X=I) double perovskites have garnered the attention of numerous researchers. These lead-free double perovskites demonstrate good stability and appropriate band gaps, making them viable candidates for future solar cell applications. As one of the most extensively studied vacancy-ordered halide double perovskites, Cs_2_SnI_6_ is a defect version of the three-dimensional (3D) CsSnI_3_ perovskite with half of the Sn atoms in the Sn-centered octahedron absent. In the fabrication of solar cells, the inefficiency of Cs_2_SnI_6_ is a problem. Cs_2_SnI_6_-based solar cells have an efficiency of approximately 1.5%, while Cs_2_SnI_4_Br_2_-based solar cells with mixed halides have an efficiency of 2.025%.

Cs_2_PtI_6_ is one of the intriguing materials in the A_2_BX_6_ class. Cs_2_PtI_6_ is an ideal option for PSCs due to its higher carrier mobility, higher absorption coefficient (4 × 10^5^ cm^−1^), narrow band gap (1.37 eV), and dynamically stable structure.^22^ With a tolerance factor of 0.97, it is able to have a highly consistent cubic structure, hence increasing its stability. It is stable under harsh conditions, including high temperatures, UV light, and high humidity.^23^ It could serve as an appropriate substitute for perovskite-containing lead due to its oxidation resistance, high atomic number, and stability beyond non-toxicity. Cs_2_PtI_6_ is a highly advantageous photovoltaic material due to all of these qualities.

Unfortunately, systematic simulation and device modeling for the properties of Cs_2_PtI_6_ perovskite, which are essential for their optoelectronic devices, are rarely investigated, resulting in subpar device performance. To further improve the performance of Cs_2_PtI_6_-based PSCs, it is required to construct a band structure that minimizes charge recombination while enhancing carrier separation and transport. For this objective, device simulation is used to gain a comprehensive understanding of the relationship between the properties of materials and performance parameters. Using SCAPS-1D, a novel n-i-p device architecture (FTO/SnO_2_/Cs_2_PtI_6_/MoO_3_/C) is theoretically investigated in this work. Theoretical investigations were conducted to optimize the performance structure of the device by varying the thickness of the perovskite and selective charge transport layer, the absorber and interface defect density, the operating temperature, the back contact, the series and shunt resistance, respectively. At 300 K, the improved device demonstrated an outstanding PCE of 23.52%, which is greater than the previously reported figures. These findings will provide theoretical recommendations for boosting the performance of Cs_2_PtI_6_-based PSCs and pave the way for the broad adoption of environmentally friendly and stable perovskites.

## 2 Theoretical simulation

For our numerical simulations, we used SCAPS-1D software version 3.3.10. In SCAPS-1D a total of seven layers, along with different front and back contacts, could be employed as input. One can analyze *J*–*V* characteristics, ac characteristics (*C*–*f* and *C*–*V*), device efficiency (*η*), open circuit voltage (*V*_oc_), fill factor (FF), short circuit current density (*J*_sc_), spectral response (QE) of device using SCAPS-1D. The simulations are based on three equations: Poisson's eqn (1) and continuity equation for holes and electrons eqn (2):1
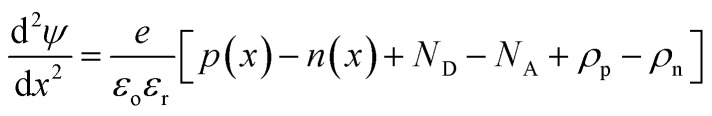
where *ψ* is electrostatic potential, *n* and *p* are electron and hole concentrations, *ε*_o_ is vacuum and *ε*_r_ is relative permittivity, *N*_D_ and *N*_A_ are donor and acceptor doping density, *ρ*_n_, *ρ*_p_ are electrons and holes distribution,2
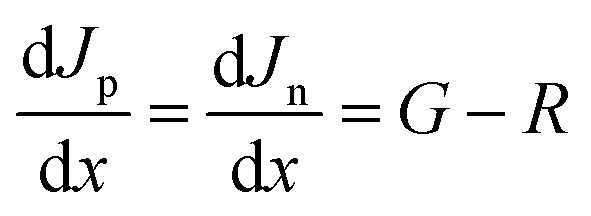
where *G* is generation rate and *R* is recombination rate, *J*_p_ and *J*_n_ are holes and electron current densities.

Carrier transport occurs according to drift and diffusion equations:3
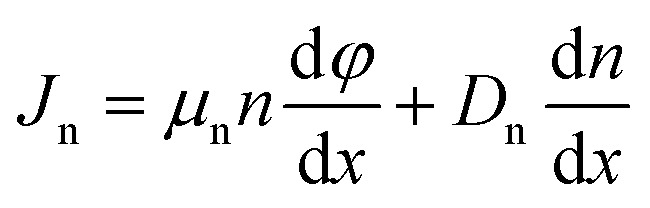
4
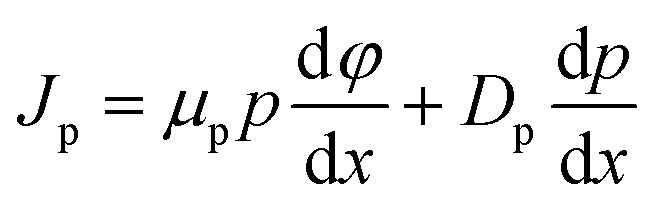


**Fig. 1:**
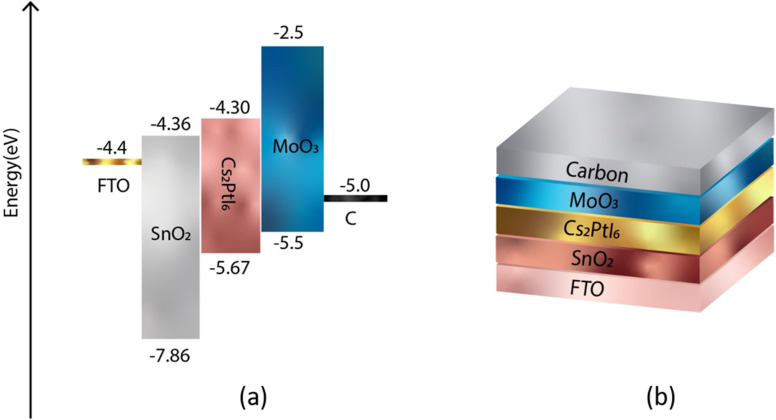
(a) Energy level diagram of FTO, ETL (SnO_2_), perovskite (Cs_2_PtI_6_), HTL (MoO_3_), back contact C (b) a schematic of device structure of n-i-p FTO/SnO_2_/Cs_2_PtI_6_/MoO_3_/C under AM 1.5 spectra, constant illumination 1000 W m^−2^, working temperature 300 K, shunt resistance 4200 Ω cm^2^ and series resistance 1 Ω cm^2^.

**Fig. 2:**
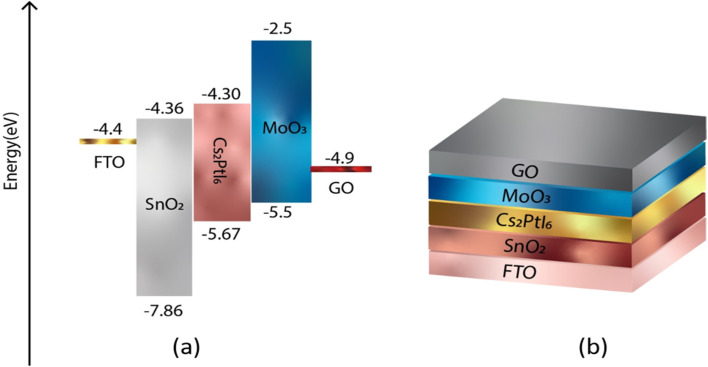
(a) Energy level diagram of FTO, ETL (SnO_2_), perovskite (Cs_2_PtI_6_), HTL (MoO_3_), back contact GO (b) a schematic of device structure of n-i-p FTO/SnO_2_/Cs_2_PtI_6_/MoO_3_/GO under AM 1.5 spectra, constant illumination 1000 W m^−2^, working temperature 300 K, shunt resistance 4200 Ω cm^2^ and series resistance 1 Ω cm^2^.

In order to achieve a higher level of efficiency, the hole transport layer (HTL) and electron transport layer (ETL) must have band gap edges that correspond with the VBM and CBM of the active layers. Fig. 1 and 2 illustrate the band gap alignment of MoO_3_, SnO_2_, and Cs_2_PtI_6_, as well as the back and front device contacts. The lowest unoccupied molecular orbital (LUMO) of SnO_2_ (ETL) is in excellent alignment with the conduction band of Cs_2_PtI_6_. Likewise, the highest occupied molecular orbital (HOMO) of MoO_3_ (HTL) is well-aligned with the valence band level of an absorbing material.

Table 1 displays the input parameters for FTO, SnO_2_, Cs_2_PtI_6_, and MoO_3_ derived from the literature. The work functions of front contact (FTO) and back contact (carbon) are 4.4 eV and 5.0 eV, respectively. Thermal velocity of both holes and electrons is 1 × 10^7^ (at 300 K). For all defects, defect type is taken as neutral, characteristic energy is 1.0 eV, energetic distribution is single. Defect density for both interfaces is 1 × 10^13^, capture cross-section of electrons and holes is 1 × 10^18^. All simulations were performed under AM 1.5 spectra, constant illumination 1000 W m^−2^, working temperature 300 K, shunt resistance 4200 Ω cm^2^ and series resistance 1 Ω cm^2^.

**Table 1:** Input parameters for materials used in the device architecture FTO/SnO_2_/Cs_2_PtI_6_/MoO_3_/C

Input parameters	FTO		*n*-SnO_2_	Cs_2_PtI_6_	*n*-MoO_3_
Thickness, *d* (nm)	500		10	300	30
Band gap, *E*_g_ (eV)	3.5		3.5	1.37	3
Electron affinity, *χ*	4		4	4.3	2.5
Permittivity, *ε*_r_	9		9	4.8	12.5
Electron mobility, *μ*_n_ (cm^2^ V^−1^ s^−1^)	20		20	62.6	25
Hole mobility, *μ*_p_ (cm^2^ V^−1^ s^−1^)	10		10	62.6	100
Effective density of states at CB, *N*_c_ (cm^−3^)	2.2 × 10^18^		2.2 × 10^17^	3.0 × 10^14^	2.2 × 10^18^
Effective density of states at VB, *N*_v_ (cm^−3^)	1.8 × 10^19^		2.2 × 10^16^	1.0 × 10^17^	1.8 × 10^19^
Density of n-type doping, *N*_D_ (cm^−3^)	1 × 10^19^		1 × 10^18^	1 × 10^12^	0
Density of p-type doping, *N*_A_ (cm^−3^)	0		0	1 × 10^15^	1 × 10^−18^
Defect density, *N*_t_ (cm^−3^)	—		1 × 10^15^	1 × 10^17^	1 × 10^15^
Capture cross-section electron (cm^2^)	—		2 × 10^−14^	1 × 10^−15^	1 × 10^−15^
Capture cross-section holes (cm^2^)	—		2 × 10^−14^	1 × 10^−15^	1 × 10^−15^
Reference	24		25	23 and 26	27

## 3 Result and discussion

### 3.1 Effect of HTL thickness

**Fig. 3:**
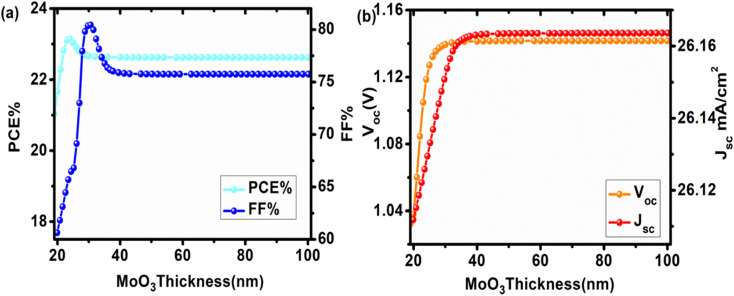
Influence of varying MoO_3_ (HTL) thickness (20–100 nm) on device performance of configuration FTO/SnO_2_/Cs_2_PtI_6_/MoO_3_/C PSC (a) comparison of PCE and FF, (b) comparison of *V*_oc_ and *J*_sc_.

To ensure that the same number of charge carriers reach terminals instantaneously with a low recombination probability, the thickness of the HTL is generally higher than that of the electron transport layer. In general, the recombination rate increases as the HTL's thickness decreases. Fig. 3 illustrates the impact of HTL thickness on device properties. Here, MoO_3_ thickness ranged from 20 to 100 nm. Both *V*_oc_ and *J*_sc_ increased with increasing MoO_3_ thickness (Table S1†), reaching saturation at 32 nm with a maximum of 26.163 mA cm^−2^ and 1.14 V, respectively. Both PCE and FF increased with increasing thickness until reaching a maximum value, after which they reduced till reaching a constant value.

When the HTL thickness is too thin, current leakage and low shunt resistance can occur, resulting in a lower FF.^28^ In our case, it decreased after reaching the highest FF value of 80.44% at 22 nm. It could be because increasing HTL thickness increased series resistance, causing the FF to drop to 75.74% at 60 nm before remaining unchanged.^28^ At 24 nm, the maximum efficiency of 23.13%, *V*_oc_ of 1.118 V, *J*_sc_ of 26.158 mA cm^−2^, and FF of 79.105% was obtained. Thus, the optimum HTL thickness of 24 nm was utilized for our subsequent devices.

### 3.2 Effect of absorbing layer thickness

**Fig. 4:**
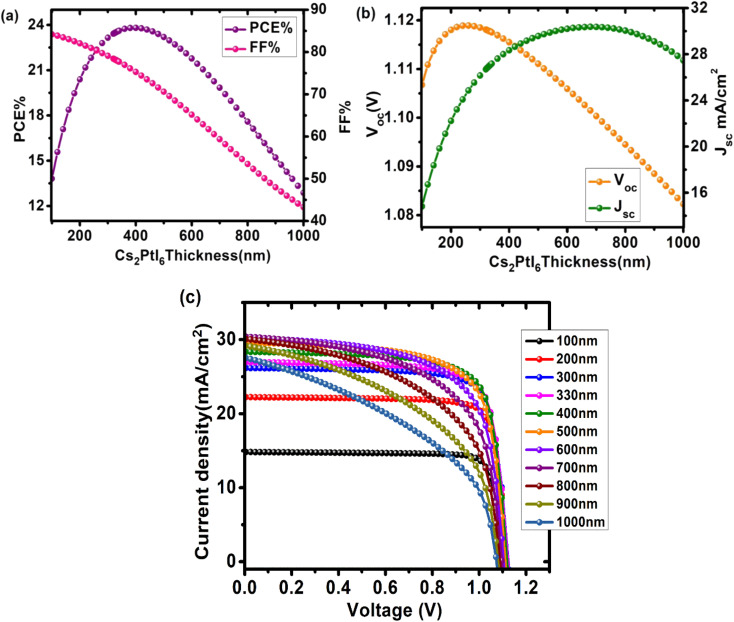
Impact of changing Cs_2_PtI_6_ (absorber layer) thickness (100–1000 nm) on performance of cell configuration FTO/SnO_2_/Cs_2_PtI_6_/MoO_3_/C PSC (a) PCE and FF, (b) *V*_oc_ and *J*_sc_, (c) comparison of *J*–*V* curves at different Cs_2_PtI_6_ thicknesses.

In designing a solar cell, the thickness of the perovskite is crucial because it directly affects the device parameters. Because electron–hole pair generation occurs in the absorbing layer, increasing thickness increases incident light absorption and generates more electron–hole pairs. This increases the device's PCE, but only up to a certain point, after which efficiency degrades. In this case, the perovskite thickness should not exceed the carrier diffusion length; otherwise, recombination and back contact recombination density would increase. The influence of absorbing layer thickness on PCE, *J*_sc_, *V*_oc_, and FF is shown in Fig. 4 and Table S2†. Here, the Cs_2_PtI_6_ thickness was varied from 100 nm to 1000 nm. *J*_sc_ increased as perovskite thickness increased. Because Cs_2_PtI_6_ has a higher absorption coefficient (4 × 10^5^ cm^−1^),^22^ increasing thickness allows more light to be absorbed, resulting in more electron–hole pair generation. Although the length of charge carrier diffusion in these perovskites is also longer, these electron–hole pairs can reach the corresponding electrodes to generate power.^29^ The *J*_sc_ at 680 nm reached a maximum of 30.37 mA cm^−2^ before decreasing. These findings are consistent with previously reported data.^30^

PCE also increased as the thickness increased, but only up to 400 nm, where the maximum efficiency of 23.8% is observed, and then it decreased. As a result of a higher absorption coefficient, a higher number of charge carriers are generated, resulting in the maximum possible increase in efficiency. Following this optimal thickness, PCE decreased. Despite the higher level of electron and hole generation, the perovskite thickness exceeds the carrier diffusion length of electrons and holes, resulting in increased recombination rates and decreased efficiency. When thickness increases, so do pinholes, cracks, and traps, resulting in a decline in PCE.^29^

*V*_oc_ increased with perovskite thickness until it reached a maximum of 1.11 V at 260 nm, after which it dropped precipitously. *V*_oc_ is defined as:
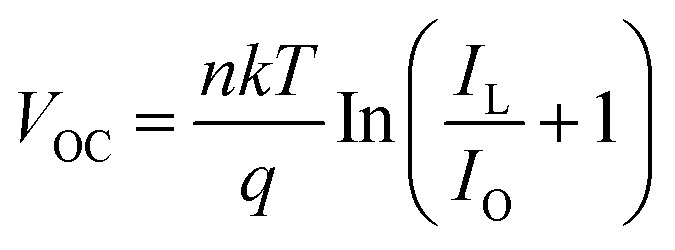
where *n* is an ideality factor, *q* is the electrical charge, *T* is temperature, *k* is the Boltzmann constant, *I*_o_ is the dark saturation current, and *I*_L_ is light generated current. *V*_oc_ is affected by cracks, pinholes, and other layer defects and is dependent on surface morphology. The absorber layer is relatively thinner during the early increase in *V*_oc_, resulting in a lower recombination rate. Furthermore, as the length of the diffusion carrier increases, so does the value of the dark saturation current. This slower recombination rate eventually leads to a higher concentration of carriers, which raises the light-generated current *I*_L_. However, as the thickness of the absorbing layer continues to increase, the recombination rate, along with *I*_o_, increases, causing the *V*_oc_ to fall abruptly and squarely.^29^ The FF relates to charge route resistance and represents the efficiency with which holes and electrons transit the cell without loss.^31,32^ As the perovskite's thickness increased, the fill factor decreased. FF decreased from 78.3% at 100 nm to 43.29% at 1000 nm as the thickness increased.

### 3.3 Effect of ETL thickness

**Fig. 5:**
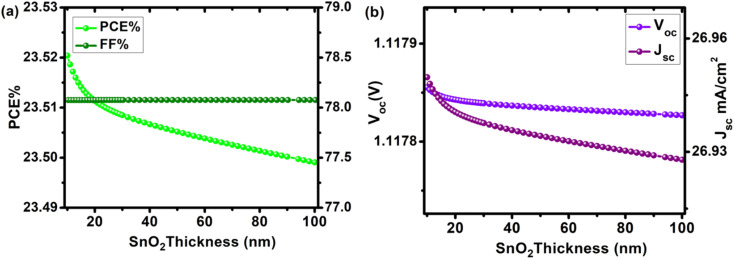
Device performance of configuration FTO/SnO_2_/Cs_2_PtI_6_/MoO_3_/C PSC as a function of increasing SnO_2_ (ETL) thickness (10–100 nm) (a) PCE and FF, (b) *V*_oc_ and *J*_sc_.

The dependence of solar cell properties on ETL (SnO_2_) was studied by varying the thickness of the electron transport layer from 10 nm to 100 nm (Table S3 †). To prevent incident photons from being absorbed and producing electron–hole pairs in the electron transport layer, it is common to practice keeping the n-type layer (ETL) thinner than the equivalent p-type layer (HTL). ETL is also kept thinner to allow incident photons to pass through to the absorber and HTLs beneath it. The influence of ETL thickness change on cell metrics is depicted in Fig. 5. By increasing ETL thickness, no improvement in cell metrics was observed. PCE, *V*_oc_, *J*_sc_, and FF were 23.52%, 1.11782 V, 26.95 mA cm^−2^, and 78.076% at 10 nm, and 23.49%, 1.11780 V, 26.93 mA cm^−2^, and 78.078% at 100 nm, respectively. With the increase in ETL thickness, the change in *V*_oc_ was insignificant (from 1.11782 V to 1.11780 V, a loss of just 0.0017%), indicating that by varying the thickness of SnO_2_ ETL, charge carrier leakage at the interface is limited. When the thickness of a device grows, fewer electron–hole pairs are formed, and charge carrier recombination occurs, resulting in a drop in overall device efficiency. Our optimal device has an ETL thickness of 10 nm, with PCE of 23.52%, *J*_sc_ of 26.95 mA cm^−2^, *V*_oc_ of 1.1178 V, and FF of 8.076%.

### 3.4 Perovskite (absorber) layer defect density

**Fig. 6:**
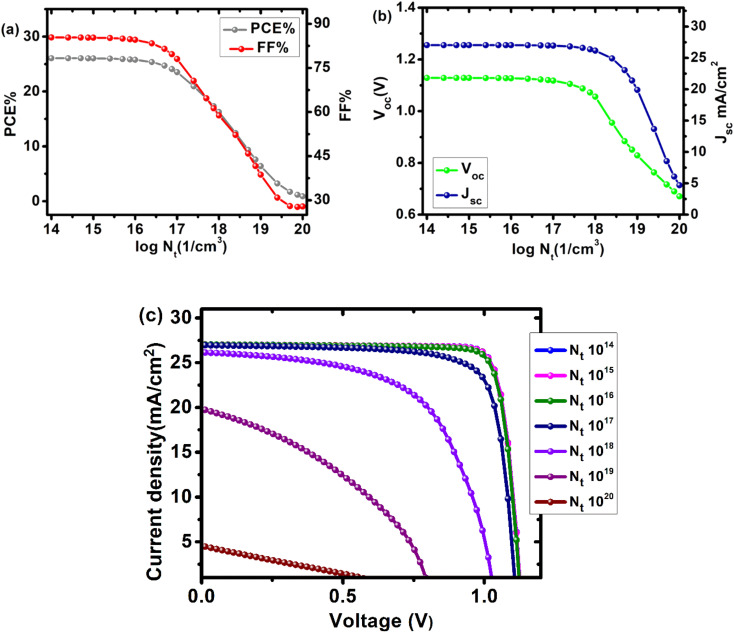
Effect of Cs_2_PtI_6_ (absorber layer) defect density (10^14^–10^20^ cm^−3^) on device parameters of cell configuration FTO/SnO_2_/Cs_2_PtI_6_/MoO_3_/C (a) PCE and FF, (b) *V*_oc_ and *J*_sc_, (c) a comparison of *J*–*V* curves of device at different absorber layer defect densities.

The optoelectronic properties of an absorbent layer are highly dependent on its preparation method, thickness, and analysis methodologies. Furthermore, defects in the system could change the optoelectronic properties. Defects were introduced into the absorbent layer to make our device appear more realistic. The defect density ranged from 10^14^–10^20^ cm^−3^. Fig. 6 depicts the effect of Cs_2_PtI_6_ defect density on cell parameters. The recombination rate increases and all cell characteristics decrease as the number of cracks and pinholes increases due to an increase in *N*_t_. An efficiency of 26% was observed with a defect density of 1 × 10^15^ cm^−3^. We chose a defect density of 1 × 10^17^ cm^−3^ for our device, which yielded an efficiency of 23.5% (Table 2). Material defects must be reduced in order for the device to be effective, and it must be smooth and free of cracks.

**Table 2:** Solar cell parameters at various Cs_2_PtI_6_ (perovskite absorber layer) defect densities (*N*_t_) for configuration FTO/SnO_2_/Cs_2_PtI_6_/MoO_3_/C

Defect density(cm^−3^)	*V*_oc_ (V)	*J*_sc_ (mA cm^−2^)	FF (%)	PCE (%)
1 × 10^14^	1.1287	27.0396	85.37	26.05
1 × 10^15^	1.1286	27.0788	85.29	26.03
1 × 10^16^	1.1275	27.0307	84.55	25.77
1 × 10^17^	1.1178	26.9497	78.08	23.52
1 × 10^18^	1.0560	26.1602	58.83	16.25
1 × 10^19^	0.8291	19.9050	38.68	6.38
1 × 10^20^	0.6708	4.6933	27.87	0.88

### 3.5 Effect of interface defect density

**Fig. 7:**
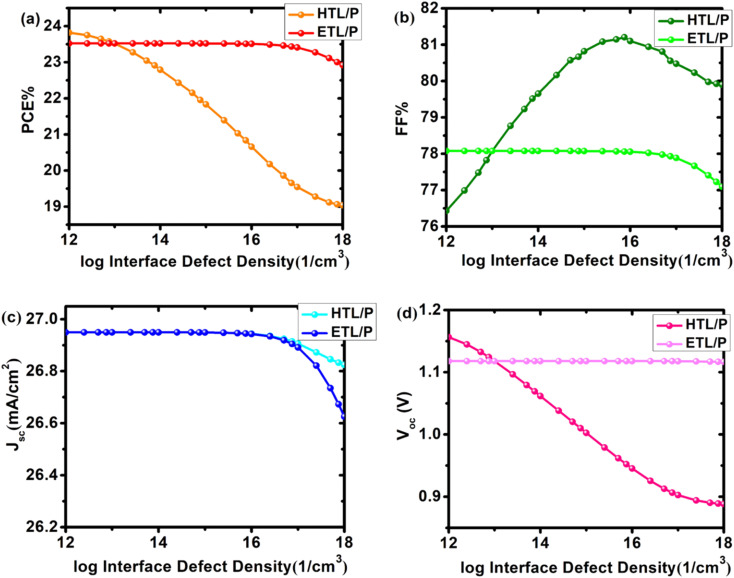
Solar cell output as a function of varying defect density *N*_t_ (10^12^–10^18^ cm^−3^) of interfaces MoO_3_/Cs_2_PtI_6_ (HTL/P) and SnO_2_/Cs_2_PtI_6_ (ETL/P) of device configuration FTO/SnO_2_/Cs_2_PtI_6_/MoO_3_/C (a) efficiency (PCE), (b) fill factor (FF), (c) short circuit current *J*_sc_ and (d) open circuit voltage *V*_oc_.

Due to a mismatch in the crystallographic structures of the ETL, HTL, and absorbing layer, interfaces with a plethora of dislocations could form, hence reducing junction quality and triggering recombination. The defect density was varied between 10^12^ cm^−3^ and 10^18^ cm^−3^ to explore the global impact of interface defect density on cell performance. Fig. 7 depicts the impact of interface defect density on cell characteristics. There was no significant change in PCE and *V*_oc_ when the defect density of the ETL/perovskite layer (SnO_2_/Cs_2_PtI_6_) was varied (Table S4 †). However, with the increase in the interface defect density of the HTL/perovskite layer (MoO_3_/Cs_2_PtI_6_), the efficiency and *V*_oc_ drastically degraded (Table S5 †). The cell's PCE decreased from 23.8% at 10^12^*N*_t_ to 19.04% at 10^18^*N*_t_. *V*_oc_ tumbled by 23%, from 1.156 V to 0.888 V.

As *N*_t_ increases, there is no discernible change in *J*_sc_. It only decreases when the prevalence of interface defects increases. By increasing the *N*_t_ of the HTL/P layer to 10^18^ cm^−3^ (from 26.94 mA cm^−2^ to 26.62 mA cm^−2^), *J*_sc_ decreased by 1.19%. Both interfaces have an effect on the fill factor. The fill factor decreased from 78.08% to 77.08% when ETL/perovskite defects increased. In the case of the HTL/perovskite layer, the defect density increased from 76.42% to an all-time high of 81.2% at *N*_t_ 10^15^ cm^−3^, before decreasing to 79.90% at defect density 10^18^ cm^−3^. We have selected a defect density of 10^13^ cm^−3^for both device interfaces.

### 3.6 Effect of temperature

**Fig. 8:**
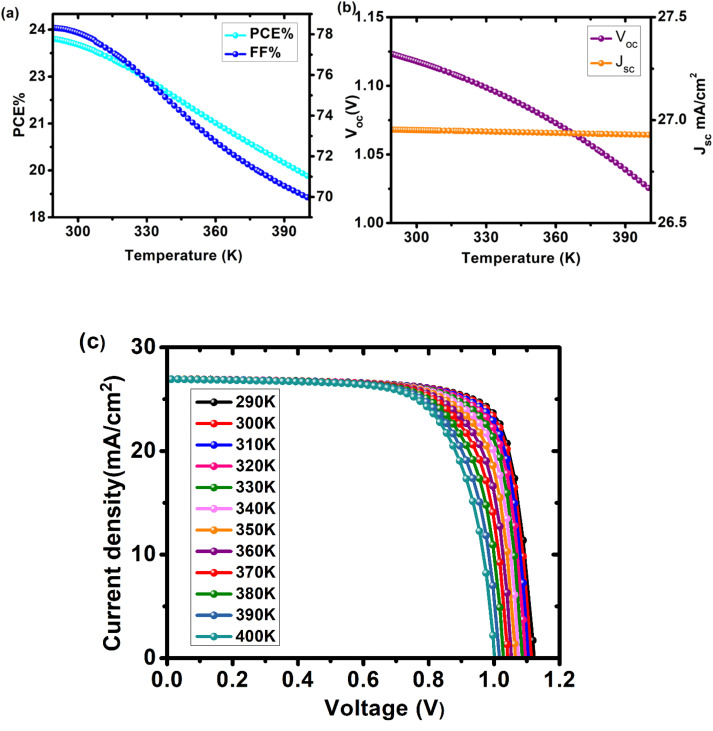
Influence of changing operating temperature (290–400 K) on parameters of cell configuration FTO/SnO_2_/Cs_2_PtI_6_/MoO_3_/C (a) PCE and FF, (b) *V*_oc_ and *J*_sc_, (c) a comparison of *J*–*V* curves of device at different temperatures.

Temperature has a direct effect on hole mobility, electron mobility, and carrier concentration, all of which affect cell performance.^33^ The device's performance was investigated at temperatures ranging from 290 K to 400 K. Eqn (5) shows the effect of temperature on *V*_oc_:^34^5
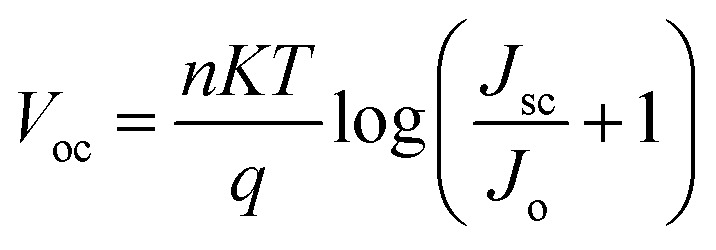
where *V*_oc_ is open circuit voltage, *K* is Boltzmann constant, *q* is the electronic charge, *n* is ideality constant, *T* is temperature, *J*_o_ is reverse saturation current and *J*_sc_ is current density. It has been observed that with the increase in the operating temperature, reverse saturation current density increases, and *V*_oc_ decreases exponentially.^35^Fig. 8 and Table S6† depict the effect of temperature on device performance. All cell metrics degraded with each increase in temperature. *V*_oc_ decreased from 1.12295 V at 290 K to 1.02557 V at 400 K. The FF decreased from 78.31% to 69.98%. There was no discernible change in the device's current density. It decreased by 0.09% from 26.9536 to 26.9282 mA cm^−2^. The device's PCE fell from 23.7% to 19.0%. As the temperature rises, the phonons are triggered, increasing charge carrier scattering, this modifies the material's conductivity. As a result, overall performance declined. For our device, we have chosen an optimal working temperature of 300 K where PCE is 23.52%, *V*_oc_ is 1.11782 V, *J*_sc_ is 26.9519 mA cm^−2^ and FF is 78.07%.

### 3.7 Effect of resistance on device parameters

Device performance is immensely influenced by both series and shunt resistance. Series resistance is due to interfaces, back and front contact, and resistance to flow of current while R_sh_ is the aftereffect of reverse saturation current. Both high R_sh_ and low R_s_ are likely to deliver better device performance. The effect of R_s_ and R_sh_ on device parameters was studied by altering values between 0.01–50 Ω cm^2^and 10–10000 Ω cm^2^. Eqn (6) can be used to understand the effect of resistance on device performance:^34^6
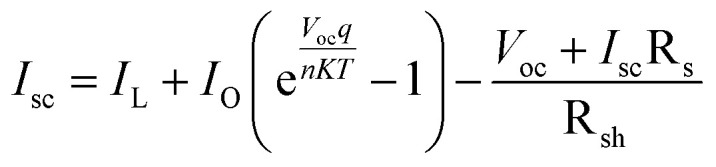
where *I*_sc_ is short circuit current, R_sh_ is shunt resistance, R_s_ is series resistance, *I*_L_ is light induced current, and *I*_O_ is reverse saturation current.

**Fig. 9:**
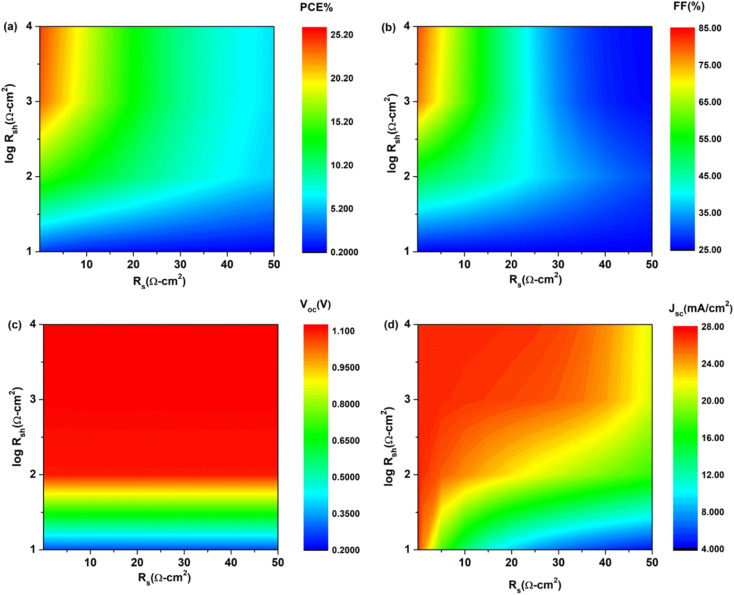
Device parameters as function of increases resistance R_s_ (0.01 to 50 Ω cm^2^) of cell configuration FTO/SnO_2_/Cs_2_PtI_6_/MoO_3_/C (a) PCE, (b) FF, (c) *V*_oc_ and (d) *J*_sc_.

According to the above equation, when series resistance increases, short circuit current will decrease. This would have a direct effect on the device's efficiency and FF. The impact of series and shunt resistance on device parameters is seen in Fig. 9. PCE and FF drop as R_s_ increases, but *J*_sc_ is only affected at higher R_s_ levels and *V*_oc_ is unaffected. At R_s_ 0.01 Ω cm^2^, a PCE high of 24.1% is measured. PCE dropped to 6.03% as the value of R_s_ increased from 0.01 to 50 Ω cm^2^. Similar to PCE, the fill factor decreased from 79.9% to 25.57% (Table 3). The initial increase in resistance had little effect on the *J*_sc_, but at 50 Ω cm^2^, it decreased from 26.959 mA cm^−2^ to 21.068 mA cm^−2^. In case of R_sh_, both PCE and FF increased with an increase in R_sh_ but only at low R_s_, *V*_oc_ isn't much affected by R_sh_ while *J*_sc_ increased with increase in R_sh_. These findings are consistent with those of other researchers. Hence, for improved device performance, the series resistance should be kept low while shunt resistance should be kept high.

**Table 3:** Solar cell parameters at different values of series resistance R_S_ (Ω cm^2^) for configuration FTO/SnO_2_/Cs_2_PtI_6_/MoO_3_/C

Series resistance (R_s_) (Ω cm^2^)	*V*_oc_ (V)	*J*_sc_ (mA cm^−2^)	FF (%)	PCE (%)
0.01	1.11770	26.959	79.99	24.10
0.1	1.11771	26.958	79.81	24.05
1	1.11782	26.949	78.07	23.52
5	1.11805	26.908	70.45	21.19
10	1.11815	26.847	61.26	18.39
25	1.11820	26.501	38.02	11.27
40	1.11827	24.528	27.12	7.44
50	1.11830	21.068	25.57	6.03

### 3.8 Quantum efficiency studies

**Fig. 10:**
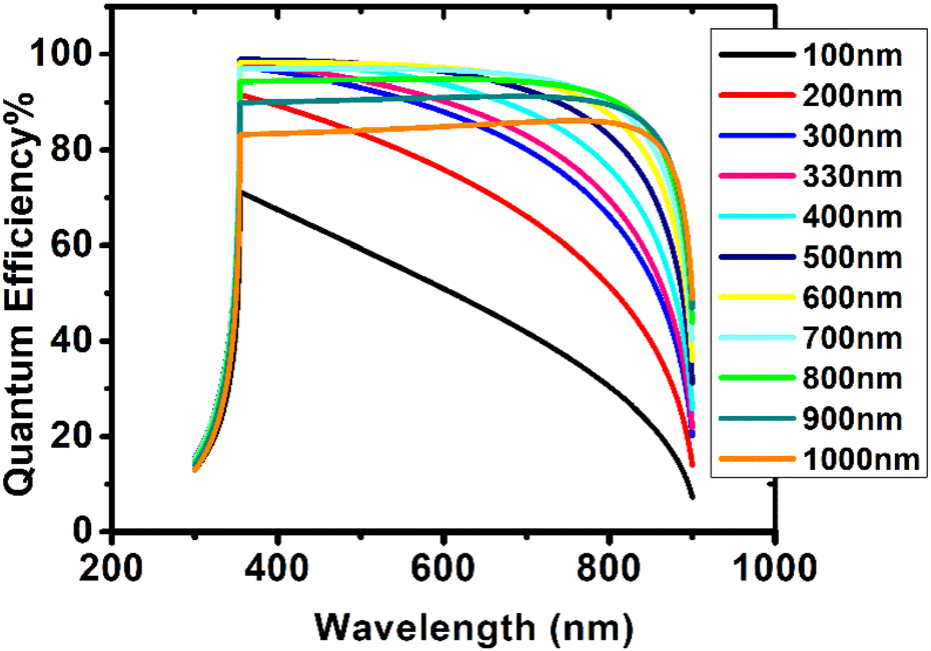
Quantum efficiency of device configuration FTO/SnO_2_/Cs_2_PtI_6_/MoO_3_/C at different Cs_2_PtI_6_ thicknesses (100–1000 nm).

Quantum efficiency (QE) is the probability that an incident photon will transfer an electron to the device's external circuit. Yet, this property is independent of the incident spectrum. Fig. 10 demonstrates the effect of absorbing layer thickness on quantum efficiency for wavelengths between 300 and 900 nm. QE increased with increasing perovskite thickness up to a maximum of 98.9% before declining. At 100 nm, the QE was 70%. At 400 nm, the concentration increased from 91.6% at 200 nm to 98.85%. A further increase in thickness did not result in a significant increase in QE, as QE at 500 nm was already 98.9%. QE decreased to 86.1% at 1000 nm as thickness rose further. In the range of 354–550 nm, high quantum efficiency was observed. As its thickness increased, its wavelength range changed towards longer values. Maximum QE was observed at the same wavelength (354.5 nm) for perovskite thicknesses between 100 nm and 600 nm, however as thickness increased, this wavelength shifted to longer wavelengths. The high QE of perovskite is due to its high absorption coefficient (4 × 10^5^ cm^−1^).^22^ This study demonstrates that Cs_2_PtI_6_ is optically active and may have additional applications in photovoltaics.

### 3.9 Effect of back contact on device parameters

**Fig. 11:**
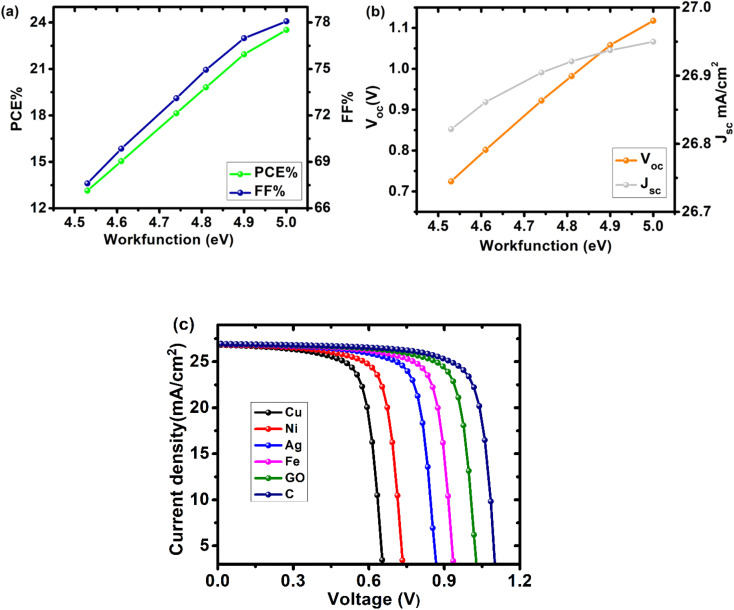
Device performance as a function of back contact work function for cell configuration FTO/SnO_2_/Cs_2_PtI_6_/MoO_3_/C (a) PCE and FF, (b) *V*_oc_ and *J*_sc_, (c) *J*–*V* curve of device with different back contacts.

**Fig. 12:**
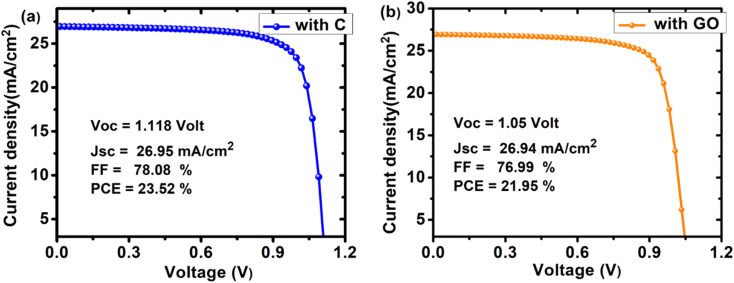
*J*–*V* curves of the optimized device having configuration (a) FTO/SnO_2_/Cs_2_PtI_6_/MoO_3_/C and (b) FTO/SnO_2_/Cs_2_PtI_6_/MoO_3_/GO.

The back contact of solar cells is essential to their performance because it absorbs electrons from the absorbing layer. For ohmic contact with the HTL or the absorber layer, a high work function is necessary. Fig. 9 illustrates the effect of the back contact work function on the device's properties. Cu, Ni, Ag, Fe, GO, and C were used as back contact materials in our device. As work function increased, efficiency grew, beginning at 13.14% with Cu as the back contact and reaching a high of 23.52% with carbon as the back contact. The other cell metrics likewise improved when the back contact's work function increased. As the work function of the back contact increases, the barrier height for charge carriers at the back contact decreases, leading to an overall improvement in cell characteristics. Table 4 compares cell properties to the work function of the back contact (Fig. 11 and 12).

**Table 4:** Solar cell parameters at different values of back contact work function (eV) for configuration FTO/SnO_2_/Cs_2_PtI_6_/MoO_3_/C

Back contact	Work function (Φ)	*V*_oc_ (V)	*J*_sc_ (mA cm^−2^)	FF (%)	PCE (%)
Cu^36^	4.53	0.72	26.822	67.60	13.14
Ni^37^	4.61	0.80	26.861	69.85	15.04
Ag^38^	4.74	0.92	26.905	73.11	18.14
Fe^39^	4.81	0.98	26.921	74.94	19.80
GO^40^	4.9	1.05	26.937	76.98	21.94
C^41^	5.0	1.12	26.949	78.08	23.52

## 4 Conclusion

We have reported the design optimization of an ecologically friendly, lead-free planar Cs_2_PtI_6_ solar cell using MoO_3_ as the HTL and SnO_2_ as the ETL (Table 5). Our research demonstrates that MoO_3_ (HTL), Cs_2_PtI_6_, and SnO_2_ have a considerable effect on device performance. The impact of varying the defect densities of the interface and absorber layers demonstrated that these parameters are crucial for device performance and that fewer defects are necessary for improved device performance. It was determined that series resistance had little influence on *V*_oc_ but a substantial effect on PCE, FF, and *J*_sc_. Effect of shunt resistance showed that both PCE and FF increase with an increase in R_sh_ (at low R_s_), V_oc_ isn't much affected by R_sh_ while *J*_sc_ increases with an increase in R_sh_. In addition, the effect of temperature on the device's functionality revealed that lower temperatures led to improved performance. The material's spectral response revealed that it was active. Cu, Ni, Ag, Fe, Go, and C were employed as back contacts, with C's work function of 5.0 eV being the best. The optimal device was the n-i-p device with the structure FTO/SnO_2_/Cs_2_PtI_6_/MoO_3_/C with a PCE of 23.52% (*V*_oc_ of 1.118 V, *J*_sc_ of 26.95 mA cm^−2^, FF of 78.08%).

**Table 5:** Performance comparison of our work and preceding work on Cs_2_PtI_6_ based PSCs

Device architecture	*V*_oc_ (V)	*J*_sc_ (mA cm^−2^)	FF (%)	PCE (%)	Experimental/simulation	Reference
ITO/SnO_2_/Cs_2_PtI_6_/spiro-OMeTAD/Au	0.73	1.20	82.00	0.72	Experimental	23
FTO/CdS/Cs_2_PtI_6_ with EDA/carbon/Cu	1.07	19.84	65.03	13.88	Experimental	22
FTO/CdS/Cs_2_PtI_6_/carbon/Cu	1.20	20.20	41.51	10.06	Experimental	22
FTO/ZnO/Cs_2_PtI_6_/MoO_3_/Cu	1.3856	16.1070	75.54	16.85	Simulation	26
FTO/ZnO/Cs_2_PtI_6_/MoO_3_/C	1.4105	16.1122	90.01	20.45	Simulation	26
FTO/CdS/Cs_2_PtI_6_/MoO_3_/Cu	1.11	20.14	61	13.9	Simulation	30
FTO/CdS/Cs_2_PtI_6_/Cu_2_O/Cu	1.1	20.4	62	14.2	Simulation	30
FTO/CdS/Cs_2_PtI_6_/CuI/Cu	1.12	20.13	60	13.7	Simulation	30
FTO/ICZSO/ Cs_2_PtI_6_/Cu_2_O/C	1.13	22.2	59.2	14.8	Simulation	30
FTO/ZnSe/Cs_2_PtI_6_/Cu_2_O/C	1.12	22.3	58	14.7	Simulation	30
FTO/WS_2_/Cs_2_PtI_6_/Cu_2_O/C	1.1	28.1	52.4	16.3	Simulation	30
**FTO/SnO**_**2**_ **/Cs**_**2**_ **PtI**_**6**_ **/MoO**_**3**_ **/GO**	**1.05**	**26.94**	**76.99**	**21.95**	**Simulation**	**This work**
**FTO/SnO**_**2**_ **/Cs**_**2**_ **PtI**_**6**_ **/MoO**_**3**_ **/C**	**1.118**	**26.95**	**78.08**	**23.52**	**Simulation**	**This work**

## Conflicts of interest

There are no conflicts to declare.

## Supplementary Material

RA-013-D3RA04176J-s001
