# Supplementary material for: Numerical simulation of lead-free vacancy ordered Cs2PtI6 based perovskite solar cell using SCAPS-1D
Source: RSC Adv. 2023 Aug 1;13(33):23211–22. doi: 10.1039/d3ra04176j (PMC10392039; doi:10.1039/d3ra04176j)
Supplement: RA-013-D3RA04176J-s001 [file RA-013-D3RA04176J-s001.pdf]

Electronic Supplementary Material (ESI) for RSC Advances.
This journal is © The Royal Society of Chemistry 2023

Numerical simulation of lead-free vacancy ordered Cs_2PtI_6 based perovskite solar cell using SCAPS-1D

Akfeen Amjad[a†], Samina Qamar[a†], Kalsoom Fatima[a], Muhammad Sultan[b*], Zareen Akhter[a*],

[a] Department of Chemistry, Quaid-i-Azam University (QAU), Islamabad 45320, Pakistan. E-mail: zareenakhter@yahoo.com, zareen_a@qau.edu.pk

[b] Department of Physics, Kohsaar University Muree (KUM), Muree (+92) 51-9269174, Pakistan. E-mail: mssatti79@yahoo.com

Supplementary Information

S1. Effect of MoO_3 thickness (10-100 nm) on device parameters

MoO_3 thickness(nm)	V_{oc} (V)	J_{sc} (mA/cm^2)	FF (%)	PCE (%)
20	1.03804	26.14745	79.81242	21.66274
24	1.11849	26.15861	79.10589	23.14476
30	1.13943	26.16273	76.03684	22.66695
40	1.14141	26.16343	75.77095	22.62769
50	1.14163	26.16354	75.74602	22.62455
60	1.14164	26.16356	75.74401	22.62431
70	1.14165	26.16357	75.74390	22.62434
80	1.14165	26.16358	75.74386	22.62434
90	1.14165	26.16360	75.74384	22.62435
100	1.14165	26.16363	75.74383	22.62436

S2. Effect of Cs_2PtI_6 thickness (100-1000 nm) on device parameters

Cs_2PtI_6 thickness(nm)	V_{oc} (V)	J_{sc} (mA/cm^2)	FF (%)	PCE (%)
100	1.10674	14.81709	84.20089	13.80776
200	1.11805	22.22641	82.05535	20.39087
300	1.11849	26.15861	79.10591	23.14497
330	1.11782	26.94972	78.07601	23.52040
400	1.11550	28.34408	75.29937	23.80797
500	1.11107	29.57439	70.60756	23.20115
600	1.10594	30.20621	65.18559	21.77602
700	1.10035	30.36836	59.37677	19.84124

800	1.09450	30.04252	53.49356	17.58954
900	1.08850	29.11874	47.98291	15.20860
1000	1.08226	27.46980	43.29790	12.87225

S3. Effect of SnO_2 thickness (10-100 nm) on device parameters

SnO_2 thickness(nm)	V_{oc} (V)	J_{sc} (mA/cm^2)	FF (%)	PCE (%)
10	1.11782	26.94972	78.07601	23.52040
20	1.11781	26.93964	78.07601	23.51135
30	1.11781	26.93640	78.07619	23.50851
40	1.11780	26.93434	78.07628	23.50669
50	1.11780	26.93265	78.07634	23.50519
60	1.11780	26.93113	78.07640	23.50385
70	1.11780	26.92970	78.07644	23.50258
80	1.11780	26.92834	78.07648	23.50137
90	1.11780	26.92701	78.07653	23.50020
100	1.11780	26.92572	78.07656	23.49906

S4. Effect of Cs_2PtI_6 /SnO_2 interface defect density on device parameters.

Cs_2PtI_6 /SnO_2 interface defect density(1/cm^3)	V_{oc}(V)	J_{sc}(mA/cm^2)	FF (%)	PCE (%)
1×10^{12}	1.11782	26.94972	78.07603	23.52041
1×10^{13}	1.11782	26.94972	78.07601	23.52040
1×10^{14}	1.11782	26.94966	78.07582	23.52029
1×10^{15}	1.11782	26.94908	78.07392	23.51917
1×10^{16}	1.11780	26.94339	78.05526	23.50810
1×10^{17}	1.11760	26.89142	77.88737	23.40806
1×10^{18}	1.11660	26.62564	77.09278	22.91991

S5. Effect of MoO_3/Cs_2PtI_6 interface defect density on device parameters.

MoO_3/Cs_2PtI_6 interface defect density (1/cm^3)	V_{oc} (V)	J_{sc} (mA/cm^2)	FF (%)	PCE (%)
1×10^{12}	1.15633	26.94972	76.42912	23.81734
1×10^{13}	1.11782	26.94972	78.07601	23.52040
1×10^{14}	1.06173	26.94966	79.65281	22.79119
1×10^{15}	1.00249	26.94912	80.82039	21.83463

1×10^{16}	0.94543	26.94393	81.10092	20.65927
1×10^{17}	0.90275	26.90611	80.47660	19.54732
1×10^{18}	0.88832	26.83425	79.90022	19.03904

S6. Effect of temperature variation (290-400K) on device parameters

Temperature (K)	V_{oc} (V)	J_{sc} (mA/cm^2)	FF (%)	PCE (%)
290	1.12295	26.95364	78.31294	23.70195
300	1.11782	26.95159	78.07601	23.5204
310	1.11217	26.94933	77.48610	23.22262
320	1.10579	26.94705	76.73043	22.86239
330	1.09967	26.94465	75.75912	22.42562
340	1.09090	26.94222	74.70142	21.95415
350	1.08231	26.93983	73.66265	21.47668
360	1.07284	26.93748	72.72965	21.01716
370	1.06255	26.93509	71.90821	20.57862
380	1.05121	26.93278	71.1901	20.15398
390	1.03896	26.93056	70.54729	19.73767
400	1.02557	26.92862	69.98351	19.32622
